# Stress Wave Propagation in a Rayleigh–Love Rod with Sudden Cross-Sectional Area Variations Impacted by a Striker Rod

**DOI:** 10.3390/s24134230

**Published:** 2024-06-29

**Authors:** Chung-Yue Wang, Nguyen Ngoc Thang, Helsin Wang

**Affiliations:** 1 Department of Civil Engineering, National Central University, No. 300, Chungda Road, Chung-Li District, Taoyuan 32001, Taiwan; nguyenngocthang@qnu.edu.vn; 2 HCK Geophysical, 9F-3, No. 79, Section 2, Roosevelt Road, Taipei 10646, Taiwan; herschel39@gmail.com

**Keywords:** nondestructive testing, stress wave propagation, Rayleigh–Love rod, Poisson’s effect, cross-sectional area variations, transmitted and reflected stress waves

## Abstract

This paper presents an in-depth study of the stress wave behavior propagating in a Rayleigh–Love rod with sudden cross-sectional area variations. The analytical solutions of stress waves are derived for the reflection and transmission propagation behavior at the interface of the cross-sectional area change in the rod, considering inertia and Poisson’s effects on the rod material. Examples solved using the finite element method are provided to verify the correctness of the analytical results. Based on the forward analysis of Rayleigh–Love wave propagation in a rod impacted by a striker rod, an impact-echo-type nondestructive testing (NDT) method is proposed to conduct defect assessment in rod-type structural components with sudden cross-sectional area changes within a cover medium. This proposed NDT method can identify the location, extension, and cross-sectional area drop ratios of an irregular zone in the rod to be inspected.

## 1. Introduction

Stress wave propagation, particularly concerning the nondestructive testing (NDT) of structures, is an important field in engineering. The NDT method is crucial for assessing the safety, reliability, and efficiency of structures [1,2,3,4,5] because it allows for the evaluation of structures and component integrity without causing damage. This approach is particularly vital for detecting defects or corrosive damage in engineering structures [6,7,8,9,10,11,12]. Corrosive damage, often manifested as reductions in the cross-sectional area of rod-type elements, is a significant concern in structural engineering. This type of deterioration typically results from environmental exposures that accelerate the corrosion process, leading to the weakening of the material and potential structural failure. Detecting and quantifying such damage is critical for maintaining structural integrity and safety. The detection of the damage condition in rod-type structural elements embedded inside a medium is an important topic in the NDT field. Among the various theories introduced to understand the longitudinal wave propagation for the impact-echo NDT method, Rayleigh–Love rod theory [13] provides a very good model of the observed real behavior. This theory combines the effects of lateral inertia and Poisson’s ratio on the rod material, which is often neglected in the traditional model of D’Alembert [14]. Incorporating lateral inertia and Poisson’s ratio is crucial for analyzing longitudinal stress wave propagation in rods because it considerably affects wave behavior, as evidenced by the research provided by Yang et al. [15,16].

The primary goal of this study was to develop an NDT method aimed at identifying the positions of cross-sectional area changes based on the forward analysis of stress wave propagation in a rod. Exhibiting abrupt changes in the cross-sectional area of a rod often indicates damage or defects in the rod-type structural member, like corrosion and bulge. To distinguish damage or defects from the effects of wave propagation modifications, reliance on changes in the amplitude of the wave is crucial; specifically, the amplitude values of reflected waves are indicative. The larger the reflected wave, the greater the damage or defect, as it indicates a discontinuity in the rod’s cross-sectional area. This change in the waveform reveals the condition of the rod and helps identify the reasons for the discontinuities affecting the waves. The theoretical foundation of this research is grounded in Rayleigh–Love rod theory [13], which provides a comprehensive understanding of stress wave propagation in rods, particularly in the context of sudden cross-sectional changes. This analytical approach enables a detailed analysis of the complex dynamics of wave propagation, including the critical effects of lateral inertia and Poisson’s ratio.

**Figure 1:**
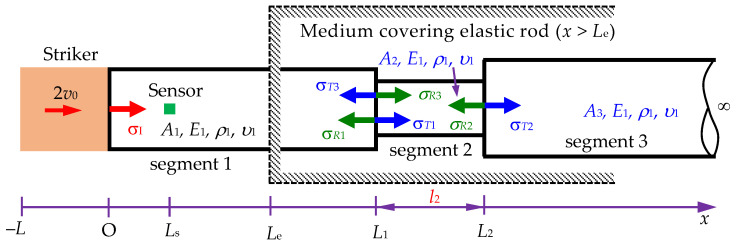
Model for determining the changing cross-sectional areas *A*_2_ and *A*_3_ and the length *L*_1_ and *L*_2_ (area reduction zone) in a cover medium (*x* > *L*_e_) based on stress wave propagation theory.

As shown in Figure 1, the model for this research was developed for using the impact-echo method to determine the changing cross-sections *A*_2_ and *A*_3_ and length *L*_1_ and *l*_2_ based on stress wave propagation signals, where *σ_I_* is the incident stress. *σ_R_*_1_ and *σ_T_*_1_ are the reflected and transmitted stresses due to *σ_I_* at *L*_1_. *σ_R_*_2_ and *σ_T_*_2_ are the reflected and transmitted stresses due to *σ_T_*_1_ at *L*_2_. *σ_R_*_3_ and *σ_T_*_3_ are the reflected and transmitted stresses due to *σ_R_*_2_ at *L*_1_. The stress propagation process begins with the impact of the striker on the semi-infinite rod, generating an incident stress (*σ_I_*) that travels along the semi-infinite rod. This incident stress (*σ_I_*) propagates to location *L*_1_, where a change in cross-sectional area causes reflected stress (*σ_R_*_1_) and transmitted stress (*σ_T_*_1_). After the stress wave passes through the cross-section change at *L*_1_, the stress wave continues to move to the changing cross-section at *L*_2_. At this location, stress superposition occurs as the transmitted stress (*σ_T_*_1_) moves toward the cross-section at *L*_2_ and the reflected stress (*σ_R_*_1_) travels back to the start of the rod. The transmitted stress (*σ_T_*_1_) will generate reflected stress (*σ_R_*_2_) and transmitted stress (*σ_T_*_2_) due to the change in cross-sectional area. The reflected wave (*σ_R_*_2_) will propagate back to position *L*_1_, generating a transmitted wave (*σ_R_*_3_) and a reflected wave (*σ_R_*_3_). *A*_1_, *A*_2_, and *A*_3_ are the cross-sectional areas of segments 1, 2, and 3 of the semi-infinite rod, respectively. *E*_1_, *ρ*_1_, and *υ*_1_ are the Young’s modulus, mass density, and Poisson’s ratio of the rod, respectively. The striker rod is moved with an impact velocity of 2*v*_0_. Then, at the observed position, an incident wave is generated, and a reflection wave travels back due to a change in the cross-sectional area. A signal is measured at a sensing site for back-calculation to determine the position and severity of a cross-sectional area change. The analysis delves into the reflection and transmission in rods with double sudden changes in cross-sectional area. Such modifications lead to alterations in the waveform during propagation. These abrupt changes are often indicative of major defects, including cracks, necking, expansion, and corrosion within the rod. This aspect is crucial because the shape and intensity of the stress waves are significantly affected at the discontinuity interface within a structure [17,18]. Previous studies [17,18] have documented the substantial influence of boundaries on wave behavior, particularly noting how sudden changes in cross-sectional area can modify wave propagation. However, these studies did not fully consider the specific effects of lateral inertia and Poisson’s ratio on stress wave propagation. Results obtained from considering lateral inertia and Poisson’s ratio show that the magnitude of stress increases, ranging from 23.84% to 30.94%, compared to results obtained without considering lateral inertia and Poisson’s ratio. This higher stress value could cause inaccurate structure analysis results if not considered. In this study, we introduce a method to identify changes in the cross-sectional area and their locations along the rod, considering the effects of lateral inertia and Poisson’s ratio. The stress values obtained are consistently higher than those calculated without considering the effects of lateral inertia and Poisson’s ratio. This is an important consideration, as exceeding allowable stress values can lead to structural damage.

## 2. Analysis of Stress Wave Propagation in a Rayleigh–Love Rod with Sudden Cross-Sectional Area Variations

### 2.1. Stress Wave Propagation in a Rod

The behavior of stress waves in a material is typically described by the wave equation. It captures the relationship between the displacement of particles within the material and the rate at which these displacements change in the spatial–temporal domains. This dynamic phenomenon is often attributed to the transmission of energy and momentum through the medium. The traditional wave equation for one-dimensional wave propagation in a rod is given by [14] as

(1) $$\frac{\partial^{2}u}{\partial t^{2}}=c_{0}^{2}\frac{\partial^{2}u}{\partial x^{2}},$$

where *u* (*x*, *t*) represents the axial (longitudinal) displacement of a point located at position *x* in the rod at time *t*, and $c_{0}$ is the wave velocity without considering Poisson’s ratio effect. Herein, wave velocity is related to the mass density *ρ* and Young’s modulus *E* of the rod by using the equation $c_{0}=\sqrt{E/\rho }$. But considering the effect of Poisson’s ratio, wave velocity (*c*) used in the stress wave propagation analysis is calculated as shown in Appendix A.

The wave equation for one-dimensional wave propagation in a rod, which takes into account the effects of lateral inertia and Poisson’s ratio as provided by Rayleigh–Love [13], is expressed as follows:

(2) $$\frac{\partial^{2}u}{\partial t^{2}}=c_{0}^{2}\frac{\partial^{2}u}{\partial x^{2}}+\upsilon^{2}\kappa^{2}\frac{\partial^{4}u}{\partial x^{2}\partial t^{2}},$$

where *u* is the axial displacement in the axial direction of stress wave propagation, *υ* is Poisson’s ratio, and *κ* is the radius of gyration of the cross-section. The influence of *κ* leads to oscillations with larger amplitudes compared to scenarios without consideration. The method for determining *κ*, related to the wave propagation equation, is detailed in Appendix B. In this theory, the stress equation can be expressed as

(3) $$\sigma =E\frac{\partial u}{\partial x}+\rho \upsilon^{2}\kappa^{2}\frac{\partial^{3}u}{\partial x\partial t^{2}}.$$

Various methods, such as the finite difference method [19,20,21,22,23,24], the finite element method (FEM) [25,26], or Laplace transform [27,28,29], can be employed to solve Equation (2). The Laplace transform, a mathematical tool that transitions functions from the time domain to the *s* domain, offers an elegant method for simplifying and solving differential equations [30]. When it is applied in solving wave propagation, this transform converts the original differential equation problem into an algebraic equation, making managing and solving easy. The solution is then converted back to the time domain using the inverse Laplace transform, which provides an accurate solution to the original wave propagation problem. Therefore, the Rayleigh–Love rod model, which considers the effects of lateral inertia, can be effectively solved with the Laplace transform. This analytical approach contributes to a detailed interpretation of the wave propagation dynamics and the consequential stress distribution, thereby providing substantial insights into the dynamic response of materials under stress wave propagation.

### 2.2. Analysis of Rayleigh–Love Rod Impacted by a Striker Rod

**Figure 2:**
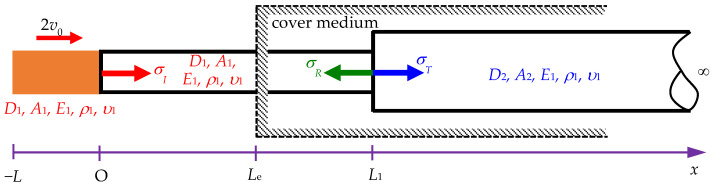
Scheme of a Rayleigh–Love rod with a sudden cross-sectional area change within a cover medium of *x* > *L*_e_, impacted by a striker rod.

Based on the results of the Laplace transform method derived from Yang et al. [16], the exact solution for the transmitted and reflected stress waves in a Rayleigh–Love rod with a sudden cross-sectional variation is derived first. Subsequently, a backward analysis method can be developed to determine the position of the changing cross-section and the ratio of the reduced area to the original area, as illustrated in Figure 2.

In Figure 2, *σ_I_*, *σ_R_*, and *σ_T_* represent the incident, reflected, and transmitted stresses, respectively. *D*_1_ is the diameter of the striker rod and rod segment 1. *D*_2_ is the diameter of rod segment 2. *A*_1_ is the cross-sectional area of the striker rod and rod segment 1. *A*_2_ is the cross-sectional area of rod segment 2. *E*_1_, *ρ*_1_, and *υ*_1_ are the Young’s modulus, mass density, and Poisson’s ratio of the rod, respectively.

**Figure 3:**
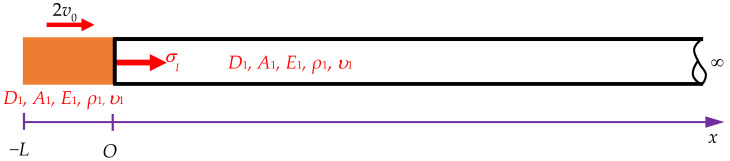
Scheme of a semi-infinite rod under the impact of a striker rod of the same material and cross-sectional area.

Yang et al. [16] focused on building an impact model based on split Hopkinson tests. The process begins with a semi-infinite rod, known as the incident bar, being struck longitudinally by a striker bar. The traditional one-dimensional theory suggests that the resulting impact generates a rectangular pulse if both rods are composed of the same material and have identical cross-sectional areas. However, the distortions of the pulses are expected on theoretical grounds and observed during experiments. Yang et al. [16] derived the analytical solutions for the longitudinal impact problems in dispersive rods. Their discussion focused on a situation where a semi-infinite Rayleigh–Love rod was impacted by a striker rod of length *L* with the same material and cross-sectional area as the Rayleigh–Love rod, as shown in Figure 3, where *σ_I_* is the incident stress, and *D*_1_ and *A*_1_ are the diameter and the cross-sectional area of the striker rod and the semi-infinite rod, respectively. *E*_1_, *ρ*_1_, and *υ*_1_ are the Young’s modulus, mass density, and Poisson’s ratio of the two rods, respectively. 2*v*_0_ is the impact velocity.

At the initial time *t* = 0, a finite striker rod moving coaxially at an impact velocity of 2*v*_0_ hits a stationary semi-infinite rod, with both rods assumed to be unstressed. Consequently, the initial conditions relating to the axial displacement *u* can be expressed as follows:

(4) $$\begin{array}{ccc} u(x,0)=0, & \frac{\partial u(x,0)}{\partial t}=2v_{0}, & -L\leq x<0 \end{array},$$

(5) $$\begin{array}{ccc} u(x,0)=0, & \frac{\partial u(x,0)}{\partial t}=0, & 0\leq x<\infty \end{array}.$$

The boundary conditions at the free end, where x=−L, and at infinity are determined by

(6) $$\begin{array}{cc} \sigma (-L,t)=0, & \left|u(\infty ,t)\right| \end{array}=0.$$

If the two rods are in tight contact in a compressed state following the collision, then the coupled continuity conditions at the contact surface *x* = 0 can be established as

(7) $$\begin{array}{cc} u(0^{-},t)=u(0^{+},t), & \sigma (0^{-},t)=\sigma (0^{+},t) \end{array}.$$

Yang et al. [16] converted the values to dimensionless quantities for easy transformation by setting $\bar{u}=\frac{u}{D_{1}}$, $\bar{x}=\frac{x}{D_{1}}$, $\bar{t}=\frac{ct}{D_{1}}$, $\bar{L}=\frac{L}{D_{1}}$, $b=\upsilon \bar{\kappa }=\upsilon \frac{\kappa }{D_{1}}$, ${\bar{v}}_{0}=\frac{v_{0}}{c}$, and $\bar{\sigma }=\frac{\sigma }{E}$. The Laplace transform and inverse techniques are employed to solve Equation (2). The result of the displacement is shown as Equation (15a) in Yang et al.’s study [16]. The stress state in a semi-infinite rod is then determined as follows:

(8) $$\bar{\sigma }\left(\bar{x},\bar{t}\right)=\frac{\partial \bar{u}}{\partial \bar{x}}+b^{2}\frac{\partial^{3}\bar{u}}{\partial \bar{x}\partial {\bar{t}}^{2}}.$$

By performing the calculation and converting the dimensionless quantities from Equation (8) into dimensional quantities, the result of stress wave propagation in the semi-infinite rod can be obtained as follows:

(9) $$\sigma_{I}\left(x,t\right)=-\frac{4\rho cv_{0}}{\pi }\int_{0}^{\frac{1}{b}}\sin\frac{L\eta }{D_{1}\sqrt{1-b^{2}\eta^{2}}}\sin\frac{\left(x+L\right)\eta }{D_{1}\sqrt{1-b^{2}\eta^{2}}}\sin\left(\frac{tc}{D_{1}}\eta \right)\left(\frac{\sqrt{1-b^{2}\eta^{2}}}{\eta }\right)d\eta ,$$

where $b=\upsilon \bar{\kappa },\;\;\;\bar{\kappa }=\frac{\kappa }{D_{1}},\;\;\;\kappa =\sqrt{\frac{I_{1}}{A_{1}}}$, η is a variable in the contour integration of the Laplace inverse transform, *ρ* is the mass density, *c* is the longitudinal wave velocity of the elastic material as expressed by Equation (A16) in Appendix A, and *v*_0_ is the impact velocity.

**Figure 4:**
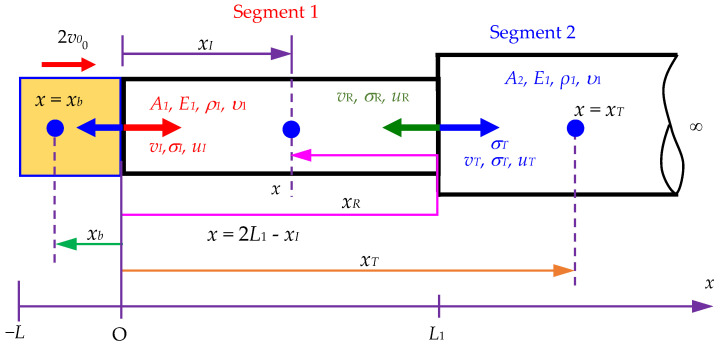
The interface between the two segments of a Rayleigh–Love rod with the same material but different cross-sectional areas.

In this study, the incident stress ($\sigma_{I}$) result of Equation (9) is considered to determine the reflected stress $\left(\sigma_{R}\right)$ and transmitted stress $\left(\sigma_{T}\right)$ in a Rayleigh–Love rod with a sudden change in cross-sectional area under the effects of Poisson’s ratio and lateral inertia. Based on the balance of forces at the interface between two segments, the total force applied by segment 1 (from the incident and reflected waves) must be equal to the total force applied by segment 2 (from the transmitted wave), as shown in Figure 4, where *v_I_*, *σ_I_*, and *u_I_* are the incident velocity, incident stress, and incident displacement of segment 1, respectively. *v_R_*, *σ_R_*, and *u_R_* are the reflected velocity, reflected stress, and reflected displacement of segment 1, respectively. *v_T_*, *σ_T_*, and *u_T_* are the transmitted velocity, transmitted stress, and transmitted displacement of segment 2, respectively. *A*_1_ and *A*_2_ are the cross-sectional areas of segment 1 and segment 2 of the semi-infinite rod, respectively. *E*_1_, *ρ*_1_, and *υ*_1_ are the Young’s modulus, mass density, and Poisson’s ratio of the two rods, respectively. *x_I_*, *x_R_*, and *x_T_* are the positions determined for the incident, reflected, and transmitted stresses in the semi-infinite rod, respectively. *x_b_* is the position-sensing stress in the striker rod. Thus, the following equation is obtained:

(10) $$A_{1}(\sigma_{I}+\sigma_{R})=A_{2}\sigma_{T},$$

where *A*_1_ and *A*_2_ are the cross-sectional areas of segment 1 and segment 2, respectively, and *σ_I_*, *σ_R_*, and *σ_T_* are the incident, reflected, and transmitted stresses, respectively.

Based on the continuity of velocity in Figure 4, the velocity at the interface of the two segments must be the same, which is expressed as

(11) $$v_{I}-v_{R}=v_{T}.$$

The relationship between stress and velocity in the rod is

(12) σ=ρcv,

where *v* is the particle velocity of the material point under stress *σ*, and *c* is the longitudinal wave velocity of the elastic material (see Appendix A) and can be determined as

(13) $$c=\sqrt{\frac{\left(1-\upsilon \right)}{\left(1+\upsilon )(1-2\upsilon \right)}}\sqrt{\frac{E}{\rho }},\;\mathrm{where}-1<\upsilon <0.5.$$

Based on Equation (12), the velocity for incident, reflected, and transmitted waves can be, respectively, presented as

(14) $$v_{I}=\frac{\sigma_{I}}{\rho_{1}c_{1}},$$

(15) $$v_{R}=\frac{\sigma_{R}}{\rho_{1}c_{1}},$$

(16) $$v_{T}=\frac{\sigma_{T}}{\rho_{2}c_{2}}.$$

Substituting Equations (14)–(16) into Equation (11) can obtain

(17) $$\frac{\sigma_{I}}{\rho_{1}c_{1}}-\frac{\sigma_{R}}{\rho_{1}c_{1}}=\frac{\sigma_{T}}{\rho_{2}c_{2}}.$$

Solving Equations (10) and (17) can obtain the reflected stress $\sigma_{R}$ and transmitted stress $\sigma_{T}$ as

(18) $$\sigma_{R1}=\frac{A_{2}\rho_{2}c_{2}-A_{1}\rho_{1}c_{1}}{A_{1}\rho_{1}c_{1}+A_{2}\rho_{2}c_{2}}\sigma_{I}=R_{1}\times \sigma_{I},$$

(19) $$\sigma_{T1}=\frac{2A_{1}\rho_{2}c_{2}}{A_{1}\rho_{1}c_{1}+A_{2}\rho_{2}c_{2}}\sigma_{I}=T_{1}\times \sigma_{I},$$

where the reflected ratio at *L*_1_ due to incident stress (*σ_I_*) is

(20a) $$R_{1}=\frac{A_{2}\rho_{2}c_{2}-A_{1}\rho_{1}c_{1}}{A_{1}\rho_{1}c_{1}+A_{2}\rho_{2}c_{2}},$$

where the transmitted ratio at *L*_1_ due to incident stress (*σ_I_*) is

(20b) $$T_{1}=\frac{2A_{1}\rho_{2}c_{2}}{A_{1}\rho_{1}c_{1}+A_{2}\rho_{2}c_{2}},$$

According to Figure 2, the stress wave propagation *σ*_1_ traveling along segment 1 is determined as

(21) $$\sigma_{1}(x,t)=\sigma_{I}(x,t)+\sigma_{R}(2L_{1}-x,t),$$

where

(22) $$\sigma_{I}\left(x,t\right)=-\frac{4\rho cv_{0}}{\pi }\int_{0}^{\frac{1}{b}}\sin\frac{L\eta }{D_{1}\sqrt{1-b^{2}\eta^{2}}}\sin\frac{\left(x+L\right)\eta }{D_{1}\sqrt{1-b^{2}\eta^{2}}}\sin\left(\frac{tc}{D_{1}}\eta \right)\left(\frac{\sqrt{1-b^{2}\eta^{2}}}{\eta }\right)d\eta ,$$

(23) $$\sigma_{R}\left(2L_{1}-x,t\right)=-\frac{4\rho cv_{0}R_{1}}{\pi }\int_{0}^{\frac{1}{b}}\sin\frac{L\eta }{D_{1}\sqrt{1-b^{2}\eta^{2}}}\sin\frac{\left(2L_{1}-x+L\right)\eta }{D_{1}\sqrt{1-b^{2}\eta^{2}}}\sin\left(\frac{tc}{D_{1}}\eta \right)\left(\frac{\sqrt{1-b^{2}\eta^{2}}}{\eta }\right)d\eta .$$

Here, $x\in \left[0,L_{1}\right]$, where *x* is the position determined for the incident and reflected stresses (see Figure 4).

The propagation of the transmitted stress propagation *σ*_2_ traveling along segment 2 is determined as

(24) $$\sigma_{2}\left(x,t\right)=\sigma_{T}=-\frac{4\rho cv_{0}T_{1}}{\pi }\int_{0}^{\frac{1}{b}}\sin\frac{L\eta }{D_{1}\sqrt{1-b^{2}\eta^{2}}}\sin\frac{\left(x+L\right)\eta }{D_{1}\sqrt{1-b^{2}\eta^{2}}}\sin\left(\frac{tc}{D_{1}}\eta \right)\left(\frac{\sqrt{1-b^{2}\eta^{2}}}{\eta }\right)d\eta .$$

Here, $x\in \left[L_{1},\infty \right]$, where *x* is the position determined for the transmitted stress.

The stress wave propagation in the striker rod is determined as follows:

(25) $$\begin{array}{cc} \sigma_{S}\left(x,t\right)=-\frac{4\rho cv_{0}}{\pi }\int_{0}^{\frac{1}{b}}\sin\frac{L\eta }{D_{1}\sqrt{1-b^{2}\eta^{2}}}\sin\frac{\left(x+L\right)\eta }{D_{1}\sqrt{1-b^{2}\eta^{2}}}\sin\left(\frac{tc}{D_{1}}\eta \right)\left(\frac{\sqrt{1-b^{2}\eta^{2}}}{\eta }\right)d\eta , & -L\leq x<0 \end{array},$$

Here, $x\in \left[-L,0\right]$, where x is the position for the determined stress wave in the striker rod.

When Poisson’s ratio is set as zero, the incident stress, reflection stress, and transmitted stress are, respectively, expressed as follows:

(26) $$\sigma_{I}\left(x,t\right)=-\frac{4\rho cv_{0}}{\pi }\int_{0}^{\infty }\sin\frac{L\eta }{D_{1}}\sin\frac{\left(x+L\right)\eta }{D_{1}}\sin\left(\frac{tc}{D_{1}}\eta \right)\left(\frac{1}{\eta }\right)d\eta =\left\{\begin{array}{ll} -\rho cv_{0}, & \frac{x}{c}<t<\frac{x+2L}{c}\\ -\frac{\rho cv_{0}}{2}, & t=\frac{x}{c},t=\frac{x+2L}{c}\\ 0, & \mathrm{otherwise} \end{array}\right.,$$

(27) $$\sigma_{R}\left(2L_{1}-x,t\right)=-\frac{4\rho cv_{0}R_{1}}{\pi }\int_{0}^{\infty }\sin\frac{L\eta }{D_{1}}\sin\frac{\left(2L_{1}-x+L\right)\eta }{D_{1}}\sin\left(\frac{tc}{D_{1}}\eta \right)\left(\frac{1}{\eta }\right)d\eta =\left\{\begin{array}{l} \begin{array}{cc} -\rho cv_{0}R_{1}, & \frac{2L_{1}-x}{c}<t<\frac{2\left(L+L_{1}\right)-x}{c} \end{array}\\ \begin{array}{cc} \begin{array}{l} \frac{-\rho cv_{0}R_{1}}{2},\\ 0, \end{array} & \begin{array}{l} t=\frac{2L_{1}-x}{c},t=\frac{2\left(L+L_{1}\right)-x}{c}\\ \mathrm{otherwise} \end{array} \end{array} \end{array}\right.,$$

(28) $$\sigma_{T}\left(x,t\right)=-\frac{4\rho cv_{0}T_{1}}{\pi }\int_{0}^{\infty }\sin\frac{L\eta }{D_{1}}\sin\frac{\left(x+L\right)\eta }{D_{1}}\sin\left(\frac{tc}{D_{1}}\eta \right)\left(\frac{1}{\eta }\right)d\eta =\left\{\begin{array}{l} \begin{array}{cc} -\rho cv_{0}T_{1}, & \frac{x}{c}<t<\frac{x+2L}{c} \end{array}\\ \begin{array}{cc} \begin{array}{l} \frac{-\rho cv_{0}T_{1}}{2},\\ 0, \end{array} & \begin{array}{l} t=\frac{x}{c},t=\frac{x+2L}{c}\\ \mathrm{otherwise} \end{array} \end{array} \end{array}\right..$$

The numerical integration provided by Yang et al. [31] was utilized to obtain the stress wave propagation in a Rayleigh–Love rod to solve Equations (21)–(25). When the stress wave propagation does not consider Poisson’s ratio and inertia effects in Equations (21)–(25), they will degenerate to Equations (26)–(28), and the analytical solution can be obtained.

## 3. Examples

### 3.1. Example 1: Study of the Stress Wave Propagation in a Rod with A_1_ ≤ A_2_

#### 3.1.1. Verification of the Analytical Solution from Equations (21)–(25) with the FEM Solution

This section presents the stress wave propagation in the rods consisting of two segments with different cross-sectional areas, where cross-sectional area *A*_2_ is greater than *A*_1_, as shown in Figure 2. First, the accuracy of Equations (21)–(25) will be confirmed by comparing the results derived from these equations and the results obtained from the FEM. The parameters of material properties and geometries of the striker rod and the segment rods used in this study are shown in Table 1.

**Figure 5:**
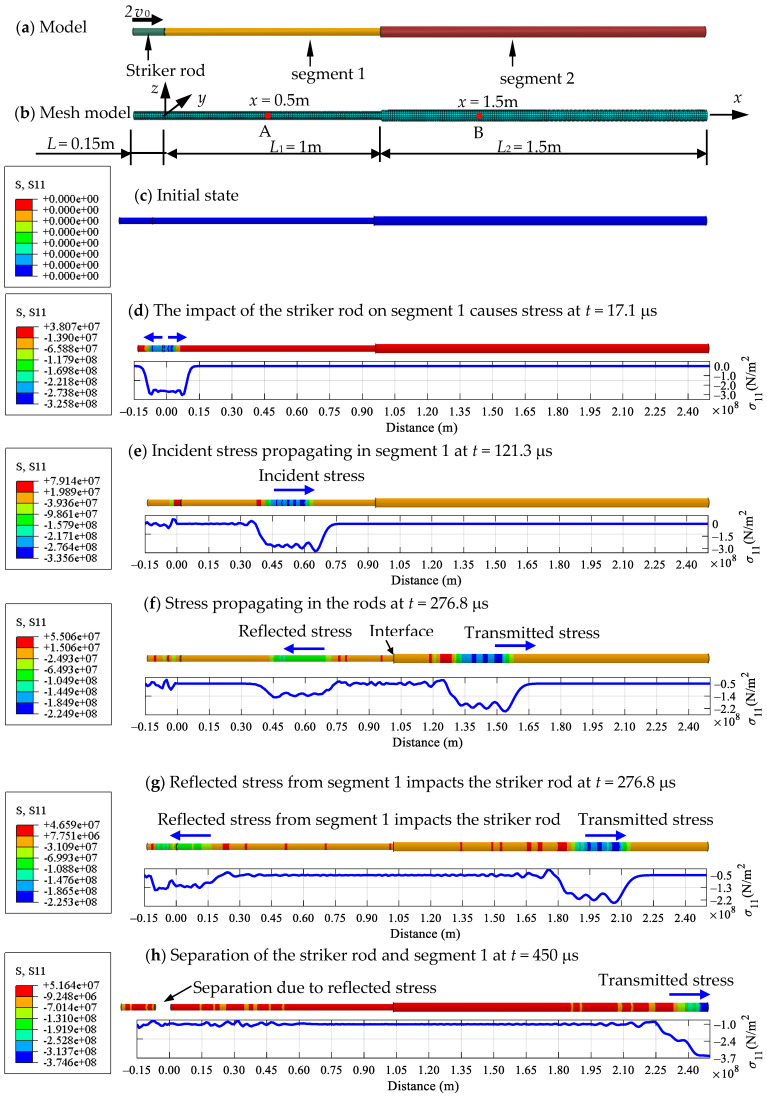
Stress wave propagating in a Rayleigh–Love rod model using solid elements for cross-sectional area ratio *A*_2_/*A*_1_ = 2 analyzed using the finite element analysis code in Abaqus.

The geometrical shape and finite element mesh of the Rayleigh–Love rod model are plotted in Figure 5a,b, and the relevant parameters used in the Rayleigh–Love rod model are shown in Table 1. The elastic material wave velocity *c* of the rod and striker materials was computed as 5782.69 m/s. In the Abaqus finite element analysis code, the solid element-type C3D8R was employed, with 10,947 nodes and 8448 elements in the model, as shown in Figure 5b. The detailed stress variations are demonstrated in Figure 5c–h at the time from the initial state to 450 μs.

**Figure 6:**
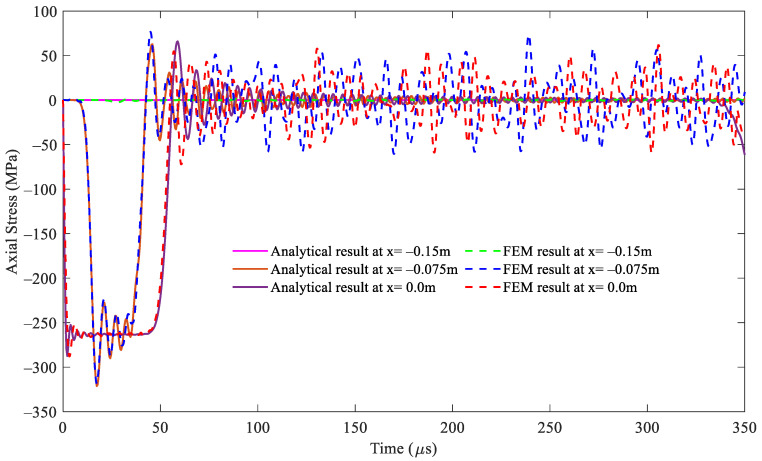
Comparison of the stress wave propagation in the striker rod between the analytical solution and FEM results for the cross-sectional area ratio *A*_2_/*A*_1_ = 2.

**Figure 7:**
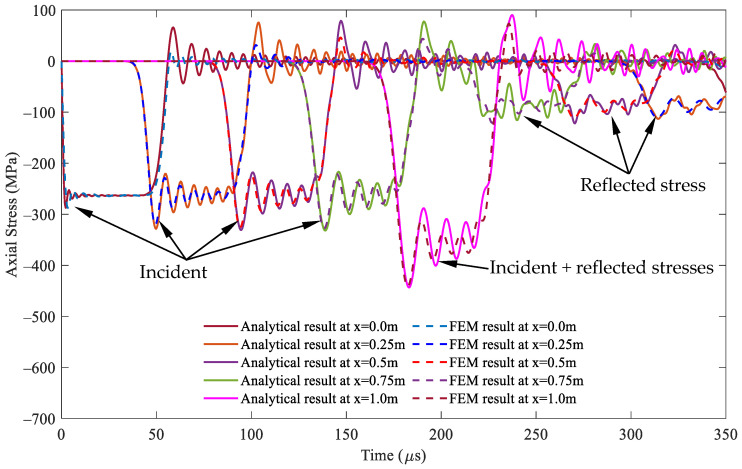
Comparison of the stress wave propagation in segment 1 between the analytical solution and FEM results for the cross-sectional area ratio *A*_2_/*A*_1_ = 2.

**Figure 8:**
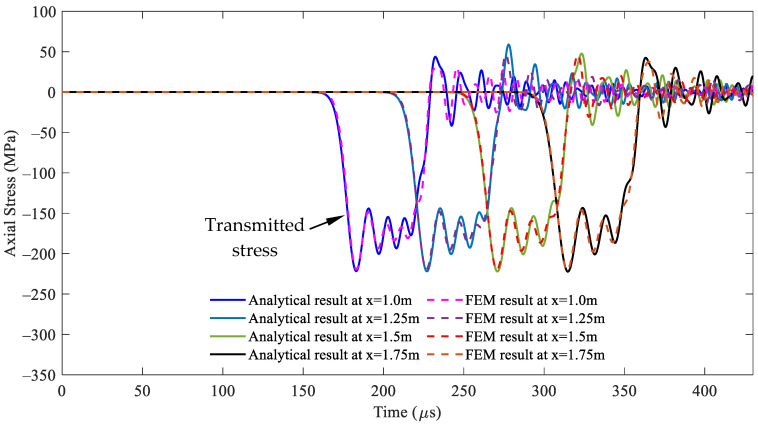
Comparison of the stress wave propagation in segment 2 between the analytical solution and FEM results for the cross-sectional area ratio *A*_2_/*A*_1_ = 2.

For the cross-sectional area ratio *A*_2_/*A*_1_ = 2, i.e., *A*_2_ = 1.41 × 10^−3^ mm^2^, the comparisons of the stress responses over time at varying positions at the striker rod, segment 1, and segment 2 between both methods are illustrated in detail in Figure 6, Figure 7 and Figure 8, respectively, through the whole wave propagation duration. A high consistency presents positive evidence for a high degree of match between the analytical solutions expressed by Equations (21)–(25) and the FEM simulation results. This verifies the correctness of the numerical algorithm [31] used to solve Equations (21)–(25) in this study.

#### 3.1.2. Investigation of Stress Wave Propagation in a Rod at Various Positions

**Figure 9:**
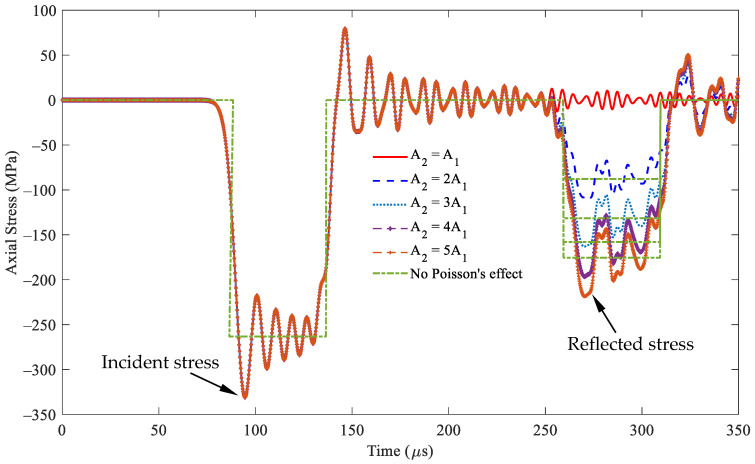
Stress wave propagation for the incident and reflected stresses in some cases of *A*_2_ ≥ *A*_1_ observed at *x* = 0.5 m (impact velocity 2*v*_0_ = 11.6 m/s).

Figure 9 presents the two types of stress responses, incident and reflected stresses, at *x* = 0.5 m, i.e., point A in Figure 5b, in segment 1, within the rod based on Equation (21). The shape of the incident stresses remains unchanged in the period of 90–140 μs, as expected. By contrast, the reflected stress in the period of 260–310 μs significantly varies with cross-sectional area *A*_2_. In particular, an increase in cross-sectional area *A*_2_ results in a corresponding increase in the magnitude of the reflected stresses. When the cross-sectional areas are equal, i.e., *A*_1_ = *A*_2_, no reflected stresses are observed, signifying the absence of reflected stresses. Moreover, the incident and reflected stresses exhibit the same compression stress responses.

Figure 9 also shows the stress responses without considering Poisson’s effect. The peak stress values of the incident stress wave are −333.24 and −263.286 MPa, respectively. This finding indicates that the stress under Poisson’s effect increases by 25.57% compared with that without Poisson’s effect. Table 2 shows that the reflected stress values range from 23.84% to 24.55% for the cross-sectional area ratio, *A*_2_/*A*_1_, varying from two to five. This finding demonstrates that Poisson’s effect has similar stress responses for different cross-sectional area ratios.

**Figure 10:**
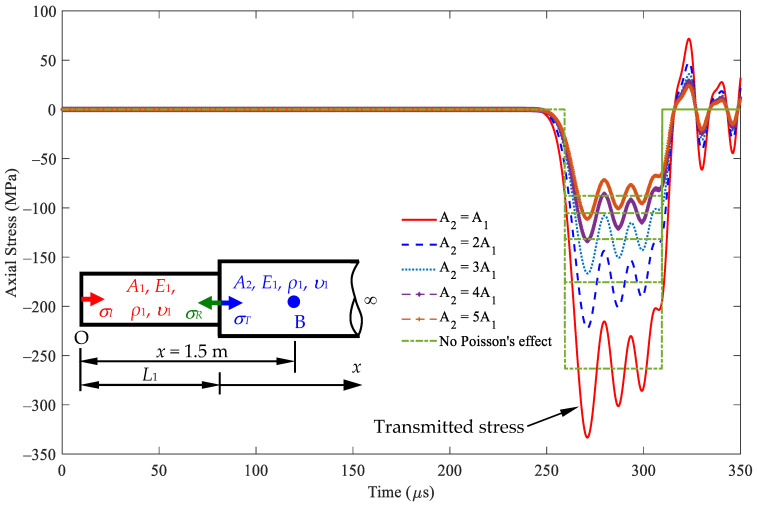
Stress wave propagation for the transmitted waves in some cases of *A*_2_ ≥ *A*_1_ observed at *x* = 1.5 m (impact velocity 2*v*_0_ = 11.6 m/s).

Figure 10 illustrates the stress responses at *x* = 1.5 m, i.e., point B in Figure 5b, in segment 2 within the rod based on Equation (24). The first peak values of this transmitted stress vary with cross-sectional area *A*_2_. The transmitted stresses are equal to the incident stress values when the cross-sectional area *A*_1_ is equal to *A*_2_. In particular, the resulting transmitted stress responses become increasingly intensive as the cross-sectional area *A*_2_ increases. Table 3 shows that the stress values have similar stress responses, with and without considering Poisson’s effect for different cross-sectional area ratios, *A*_2_/*A*_1_. The waveforms of nonzero Poisson’s ratio oscillate about the waveform of zero Poisson’s value.

**Figure 11:**
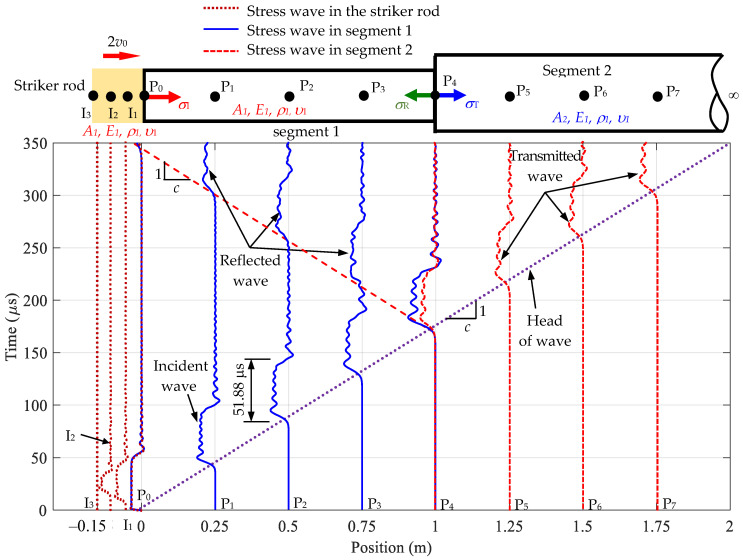
Stress wave travel time history at various positions in the striker rod and Rayleigh–Love rod with a sudden cross-sectional area variation for the incident, reflected, and transmitted waves depending on position and time.

Figure 11 illustrates the stress wave travel time history at various positions in a Rayleigh–Love rod. The stress wave propagation in the striker rod is presented at points I_1_, I_2_, I_3_, and P_0_ (left). Points P_0_ (right) to P_3_ represent the presence of the incident and reflected waves. Points P_5_ to P_7_ describe the transmitted wave in the semi-infinite rod. The dotted lines represent stress in the striker rod, the solid lines represent the incident and reflected waves, and the dashed lines correspond to the transmitted wave.

The interface of the cross-sectional area jumps off the rod, and all three types of waves (incident, reflected, and transmitted waves) are observed at P_4_. The incident and reflected waves are detected on the left side of point P_4_, whereas the transmitted wave is discernible on the right side. The oscillations of the wave, influenced by Poisson’s effect, are consistently found at each position, leading to stress values surpassing those from traditional wave equations that disregard Poisson’s effect. This finding emphasizes the pertinence of the theoretical approach in aligning closely with the actual wave propagation observed in real tests and verifies its applicability to structural analysis works.

### 3.2. Example 2: Analysis of the Signal Obtained for the Rod Where A_1_ ≥ A_2_

**Figure 12:**
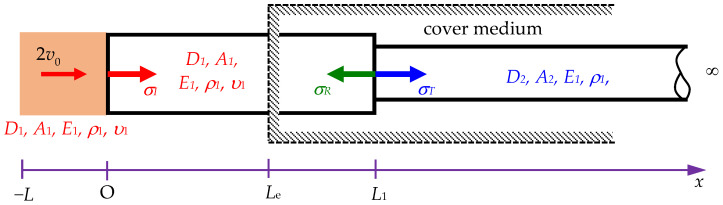
Scheme of a Rayleigh–Love rod with a sudden cross-sectional area change from large to small embedded in a cover medium of *x* > *L*_e_.

This section presents stress wave propagation where cross-sectional area *A*_2_ is less than cross-sectional area *A*_1_, as shown in Figure 12, where *σ_I_*, *σ_R_*, and *σ_T_* represent the incident, reflected, and transmitted stresses, respectively. *D*_1_ is the diameter of the striker rod and rod segment 1. *D*_2_ is the diameter of rod segment 2. *A*_1_ is the cross-sectional areas of the striker rod and rod segment 1. *A*_2_ is the cross-sectional area of rod segment 2. *E*_1_, *ρ*_1_, and *υ*_1_ are the Young’s modulus, mass density, and Poisson’s ratio of the rod, respectively. Except for the diameters of segment 2, the relevant parameters used in the Rayleigh–Love rod model are listed in Table 1. The diameters of segment 2 are set as 30, 21, 17, 15, and 13 mm instead.

**Figure 13:**
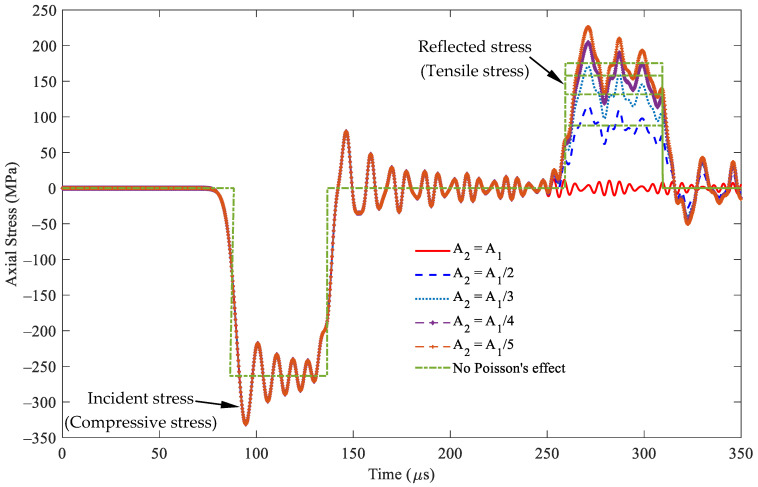
Stress wave propagation for the incident and reflected waves in some cases of *A*_2_ ≤ *A*_1_ observed at *x* = 0.5 m (impact velocity of 2*v*_0_ = 11.6 m/s).

Figure 13 depicts the stress responses and incident and reflected stresses, at *x* = 0.5 m, i.e., point A in Figure 5b, in segment 1, within the rod based on Equation (21) when cross-sectional area *A*_1_ is greater than cross-sectional area *A*_2_. The shape of the incident stresses remains invariant in the 90–140 μs period regardless of the changes in cross-sectional area *A*_2_, as expected. Conversely, the reflected stress in the 260–310 μs period significantly varies with alterations in cross-sectional area *A*_2_. If *A*_2_ is smaller than *A*_1_, this leads to intensive reflected stresses. When the cross-sectional areas are equal, i.e., *A*_1_ = *A*_2_, no reflected stress responses are observed, signifying the absence of reflected stresses. In this scenario, the incident and reflected stresses exhibit opposite stress states, indicating that the reflected wave phase is strongly affected by the cross-sectional area ratio at the interface. Table 4 shows that the stress values have similar stress responses with and without Poisson’s effect for different cross-sectional area ratios. 

**Figure 14:**
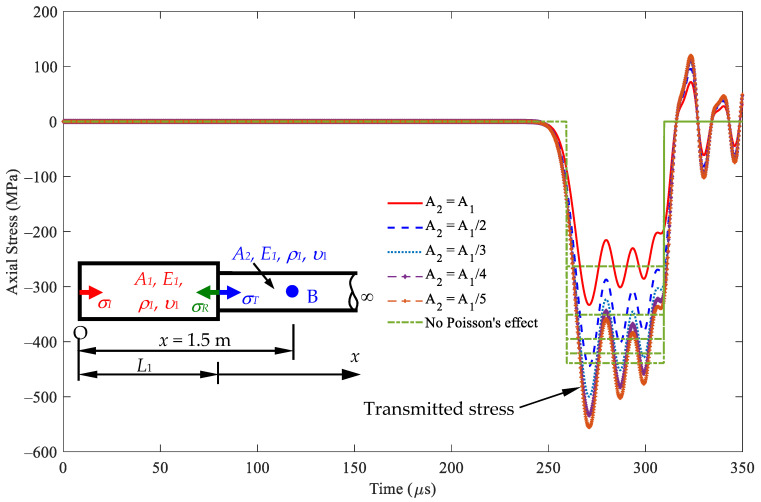
Stress wave propagation for transmitted waves in some cases of *A*_2_ ≤ *A*_1_ observed at *x* = 1.5 m (impact velocity of 2*v*_0_ = 11.6 m/s).

Figure 14 illustrates the stress responses at *x* = 1.5 m, i.e., point B in Figure 5b, in segment 2 within the rod based on Equation (24). When cross-sectional area *A*_1_ is greater than cross-sectional area *A*_2_, it leads to more intensive transmitted stresses than the incident stress. When cross-sectional area *A*_1_ is equal to *A*_2_, the transmitted stresses are equal to the incident stress values. In particular, the resulting transmitted stress responses become increasingly intensive as cross-sectional area *A*_2_ increases. Table 5 shows that the stress values have similar stress responses, *A*_2_/*A*_1_, with and without considering Poisson’s effect for different cross-sectional area ratios.

## 4. Identification of the Locations of Sudden Cross-Sectional Area Change in the Rod

### 4.1. Reflection and Transmission of Stress Wave in a Rod with Single Sudden Cross-Sectional Area Variation

#### 4.1.1. Developing a Formula to Determine the Damaged Zone

In some engineering diagnosis problems, the damaged position and the condition of the rod inside a cover medium must be identified. The impact-echo method is usually applied for this purpose. The variations in the reflected and transmitted stresses can be presented in terms of cross-sectional area ratio *A*_2_/*A*_1_ to illustrate the influence of the cross-sectional area change in the rod. The reflected stress $\sigma_{R}$ and transmitted stress $\sigma_{T}$ can be expressed as follows by letting *α* = *A*_2_/*A*_1_ and substituting it into Equations (18) and (19):

(29) $$\sigma_{R}=\frac{\alpha \rho_{2}c_{2}-\rho_{1}c_{1}}{\rho_{1}c_{1}+\alpha \rho_{2}c_{2}}\sigma_{I},$$

(30) $$\sigma_{T}=\frac{2\rho_{2}c_{2}}{\rho_{1}c_{1}+\alpha \rho_{2}c_{2}}\sigma_{I},$$

where *α* = *A*_2_/*A*_1_ is the cross-sectional area ratio.

Equations (29) and (30) show the relationship between the cross-sectional area ratio and the reflected and transmitted stress. When the cross-sectional area ratio *A*_2_/*A*_1_ approaches 0, it follows that *σ_R_* approaches −*σ_I_*, and *σ_T_* approaches 2*σ_I_*; as *A*_2_/*A*_1_ approaches ∞, it follows that *σ_R_* approaches *σ_I_*, and *σ_T_* approaches 0. 

Suppose that one knows the first peak stress values, labeled $\sigma_{I}^{p}$ as the peak incident stress and $\sigma_{R}^{p}$ as the peak reflected stress. In this case, one can determine the change in the cross-sectional area *A*_2_ from the acquired signal. From Equation (29), the cross-sectional area ratio *α* can be rewritten as

(31) $$\alpha =\frac{A_{2}}{A_{1}}=\frac{\rho_{1}c_{1}\left(\sigma_{I}^{p}+\sigma_{R}^{p}\right)}{\rho_{2}c_{2}\left(\sigma_{I}^{p}-\sigma_{R}^{p}\right)}=\frac{\rho_{1}c_{1}\left(1+\sigma_{R}^{p}/\sigma_{I}^{p}\right)}{\rho_{2}c_{2}\left(1-\sigma_{R}^{p}/\sigma_{I}^{p}\right)}.$$

**Figure 15:**
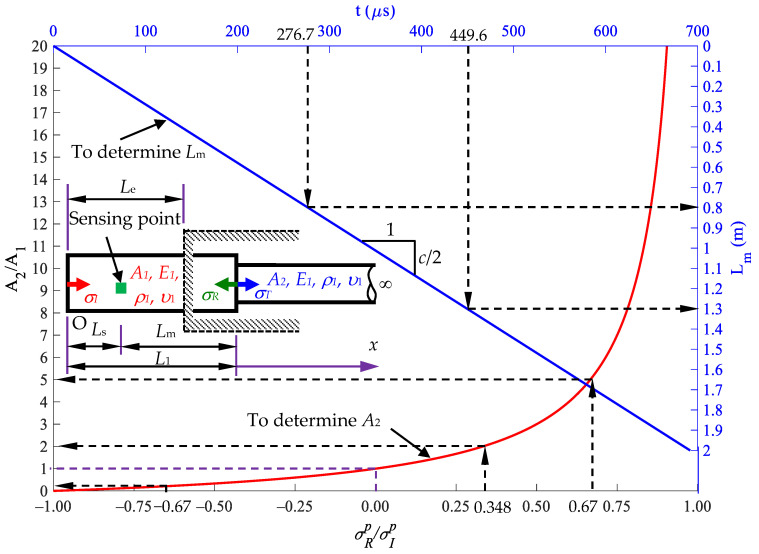
Diagram for determining the length *L*_m_ and cross-sectional area *A*_2_.

Assume that one measures the wave traveling time *t*, which is the interval of the first peak of the wave traveling from the sensing point and the first peak of the reflected wave received at the same sensing point. In this case, one can determine the length of *L*_m_ between the sensing point and the reflected interface as

(32) $$L_{m}=\frac{1}{2}tc_{1},$$

where *t* is the time duration from the first peak stress to the second peak stress, and *c*_1_ is the longitudinal wave speed of the rod material. The sensing point is located at a distance *L*_s_ from the rod end that is smaller than the embedded length *L*_e_, as shown in Figure 15.

We assume that $\rho_{1}=\rho_{2}=\rho ,\;\;\;c_{1}=c_{2}=c$. Figure 15 shows the relation curve (labeled as a red line) between the cross-sectional area ratio *A*_2_/*A*_1_ and the reflected ratio $\sigma_{R}^{p}/\sigma_{I}^{p}$ and the relation curve (labeled as a blue straight line) between the length *L*_m_ between the sensing point and the reflected interface and the wave traveling time *t*. $\sigma_{R}^{p}/\sigma_{I}^{p}=-1$ implies that *A*_2_ = 0, indicating that the reflected wave is equal in magnitude but opposite in sign to the incident wave. It also represents a free surface at position *L*_1_. $\sigma_{R}^{p}/\sigma_{I}^{p}=1$ implies that $A_{2}\to \infty$, indicating that the reflected wave is equal to the incident wave in magnitude. When $\sigma_{R}^{p}/\sigma_{I}^{p}=0$, this scenario implies that *A*_2_/*A*_1_ = 1. If no reflected wave is received, the cross-sectional area in segment 2 remains unchanged, i.e., *A*_2_ = *A*_1_. For $-1<\sigma_{R}^{p}/\sigma_{I}^{p}<0$, the cross-sectional area *A*_2_ is smaller than *A*_1_. When $0<\sigma_{R}^{p}/\sigma_{I}^{p}<1$, the cross-sectional area *A*_2_ is greater than *A*_1_. The length of *L*_1_ = *L*_m_ + *L*_s_ can be determined from Figure 15. The application in Figure 15 is demonstrated in the following subsection.

#### 4.1.2. Examples: Determination of Cross-Sectional Area *A*_2_ and Length *L*_1_ Based on Signals Obtained with Changing Cross-Sectional Area Ratios and Impact Velocities

1. Case A: Cross-sectional area ratio *A*_2_/*A*_1_ = 5

**Figure 16:**
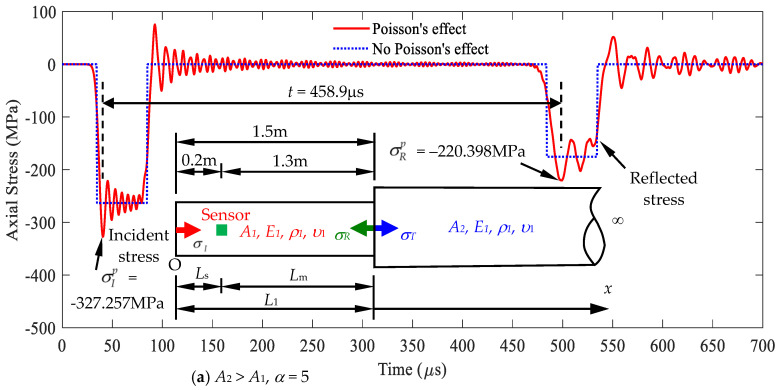

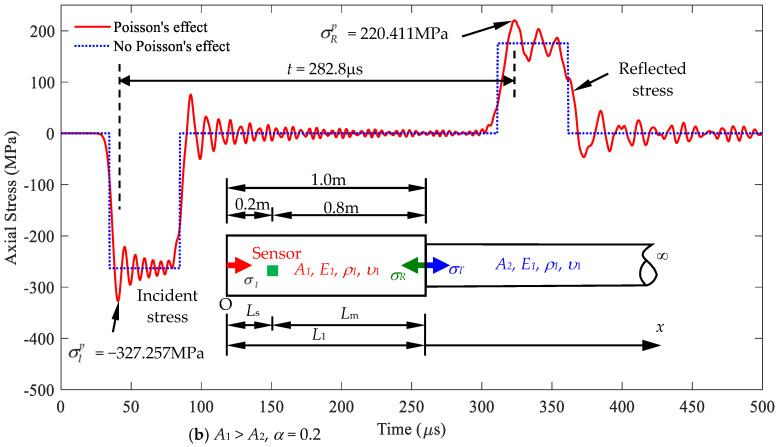
The cross-sectional area *A*_2_ and length *L*_1_ determined based on the stress signal measured at *x* = *L*_s_: (**a**) *A*_2_ > *A*_1_; (**b**) *A*_1_ > *A*_2_.

As shown in Figure 16a, Case A indicates the stress responses at the position of *x* = *L*_s_ = 0.2 m by using Equation (21). The relevant parameters used in the Rayleigh–Love rod model are identical to those used in Example 1, i.e., Table 1. The diameter of segment 2 is chosen as 67 mm. The first peak incident stress is $\sigma_{I}^{p}=-327.257\;\mathrm{MPa}$, and the first peak reflected stress is $\sigma_{R}^{p}=-220.398\;\mathrm{MPa}$, leading to a reflected ratio of $\sigma_{R}^{p}/\sigma_{I}^{p}=0.67$. Figure 16 shows the corresponding cross-sectional area ratio $\alpha =A_{2}/A_{1}=5$, which implies *A*_2_ = 5*A*_1_. The signal received by the reflected stress at time *t* is 458.9 μs, indicating that *L*_m_ = 1.3 m, from the corresponding curve in Figure 15. Given that *L*_s_ = 0.2 m is chosen, *L*_1_ is calculated as *L*_m_ + *L*_s_, equal to 1.5 m.

2. Case B: Cross-sectional area ratio *A*_2_/*A*_1_ = 0.2

As shown in Figure 16b, Case B indicates the stress responses at the position of *x* = *L*_s_ = 0.2 m by using Equation (21). The relevant parameters of materials are identical to those used in Case A except for the diameter of segment 2. The diameter of segment 2 is set as 13 mm. The first peak incident stress is $\sigma_{I}^{p}=-327.257\;\mathrm{MPa}$, and the first peak reflected stress is $\sigma_{R}^{p}=220.411\;\mathrm{MPa}$, indicating the reflected ratio of $\sigma_{R}^{p}/\sigma_{I}^{p}=-0.67$. Figure 16 shows the corresponding cross-sectional area ratio $A_{2}/A_{1}=0.2$; thus, $A_{2}=A_{1}/5$. The received signal of the reflected stress at *t* = 282.8 μs corresponds to *L*_m_ = 0.8 m in Figure 15. Given that *L*_s_ = 0.2 m is chosen, *L*_1_ is calculated as *L*_m_ + *L*_s_, equal to 1.0 m.

3. Case C: Influences of different impact velocities and striker lengths on the characteristics of response signals

**Figure 17:**
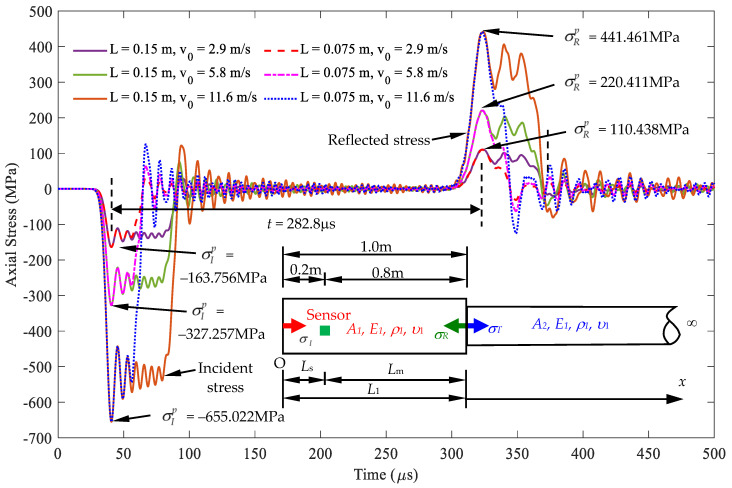
Determination of the cross-sectional area *A*_2_ and length *L*_1_ based on the stress signal measured with different velocities and striker lengths at the position of *x* = *L*_s_, *A*_1_ > *A*_2_, *α* = 0.2.

All parameters of Case C are identical to those of Case B, except for the different impact velocities and striker lengths. As shown in Table 6, the length of the striker rod varies between 0.15 m and 0.075 m, respectively. Velocities were used for the cases as 2.9 m/s, 5.8 m/s, and 11.6 m/s, respectively. The impact energy of each case that the striker applied on the rod is also listed in Table 6. Stress responses for the different cases of impact velocity and striker length combination are shown in Figure 17. This figure demonstrates that the first peak stress values varied with the impact energy of the striker input to the rod. It is noted that the time interval *t* between the first peak of the incident and reflected wave signal is fixed as *t* = 282.3 μs, and the stress ratio obtained between the cases of reflected and incident waves is the same for all cases, as calculated in Equation (33).

(33) $$\alpha =\frac{A_{2}}{A_{1}}=\frac{\rho_{1}c_{1}\left(\sigma_{I}^{p}+\sigma_{R}^{p}\right)}{\rho_{2}c_{2}\left(\sigma_{I}^{p}-\sigma_{R}^{p}\right)}=\frac{\rho_{1}c_{1}\left(1+\sigma_{R}^{p}/\sigma_{I}^{p}\right)}{\rho_{2}c_{2}\left(1-\sigma_{R}^{p}/\sigma_{I}^{p}\right)}.$$

Since all of the cases have the same arrival time interval *t* and the same first peak stress ratio $\sigma_{R}^{p}/\sigma_{I}^{p}$, they all have the same length *L*_m_ and cross-sectional area *A*_2_ as Figure 15. This case study shows that the impact-echo test method proposed in this paper provides suitable impact energy to the inspected rod and measures the response signal at a sensing point. The measurements of the impact velocity and the length of the striker are not required. The location and cross-sectional area ratio of the rod with cross-sectional area jump can be identified through the curves in Figure 15.

### 4.2. Reflection and Transmission of Stress Wave in a Rod with Double Sudden Cross-Sectional Area Variations

**Figure 18:**
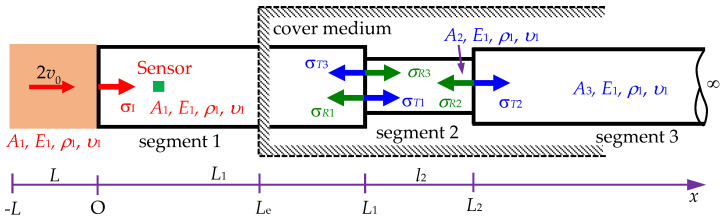
Scheme of a Rayleigh–Love rod impacted by a striker rod with double sudden cross-sectional area variations in a cover medium of *x* > *L*_e_.

This section discusses a rod with double cross-sectional area variations. Initially, the cross-section is *A*_1_, which then changes to *A*_2_ and *A*_3_ at positions *L*_1_ and *L*_2_, respectively. Using the model depicted in Figure 18, a formula is developed to determine the signal received at the sensor location. Based on this formula, changes in the cross-sectional area and the positions of these changes can be identified.

A stress wave is generated by a striker, and upon encountering a change in the cross-section, it causes a reflected wave and a transmitted wave, as described in Section 2.2. There are two locations of sudden cross-section change, as illustrated in Figure 18, where *σ_I_* is the incident stresses. *σ_R_*_1_ and *σ_T_*_1_ are the reflected and transmitted stresses due to *σ_I_* at *x* = *L*_1_. *σ_R_*_2_ and *σ_T_*_2_ are the reflected and transmitted stresses due to *σ_T_*_1_ at *x* = *L*_2_. *σ_R_*_3_ and *σ_T_*_3_ are the reflected and transmitted stresses due to *σ_R_*_2_ at *L*_1_. *A*_1_, *A*_2_, and *A*_3_ are the cross-sectional areas of segments 1, 2, and 3 of the semi-infinite rod, respectively. *E*_1_, *ρ*_1_, and *υ*_1_ are the Young’s modulus, mass density, and Poisson’s ratio of the rod, respectively. Therefore, the stress wave at the sensor location is determined as follows:

(34) $$\sigma_{S}(x,t)=\sigma_{I}(x,t)+\sigma_{R1}(2L_{1}-x,t)+\sigma_{T3}(2L_{2}-x,t),$$

where $\sigma_{I}(x,t)$ is the incident stress as shown in Equation (22); $\sigma_{R1}(2L_{1}-x,t)$ is the reflected stresses due to interface at *L*_1_ as shown in Equation (23); and $\sigma_{T3}(2L_{2}-x,t)$ is the reflected stresses obtained at the sensor position due to interface at *L*_2_ and is determined as follows:

(35) $$\sigma_{T3}(2L_{2}-x,t)=-\frac{4\rho cv_{0}R_{T3}}{\pi }\int_{0}^{\frac{1}{b}}\sin\frac{L\eta }{D_{1}\sqrt{1-b^{2}\eta^{2}}}\sin\frac{\left(2L_{2}-x+L\right)\eta }{D_{1}\sqrt{1-b^{2}\eta^{2}}}\sin\left(\frac{tc}{D_{1}}\eta \right)\left(\frac{\sqrt{1-b^{2}\eta^{2}}}{\eta }\right)d\eta ,$$

where $R_{T3}$ is the reflected ratio due to interface at *L*_1_ and *L*_2_, and the method for determining the reflection ratio is similar to that described in Section 2.2, calculated as follows:

(36) $$R_{T3}=\left(\frac{2A_{1}\rho_{2}c_{2}}{A_{1}\rho_{1}c_{1}+A_{2}\rho_{2}c_{2}}\right)\left(\frac{A_{3}\rho_{3}c_{3}-A_{2}\rho_{2}c_{2}}{A_{2}\rho_{2}c_{2}+A_{3}\rho_{3}c_{3}}\right)\left(\frac{2A_{2}\rho_{1}c_{1}}{A_{1}\rho_{1}c_{1}+A_{2}\rho_{2}c_{2}}\right).$$

Let $\alpha =\frac{A_{2}}{A_{1}},\;\beta =\frac{A_{3}}{A_{2}}$ be the cross-sectional area ratio; substituting them into Equation (36) and combining with Equation (35) obtains *β*, as follows:

(37) $$\beta =\frac{\rho_{2}c_{2}}{\rho_{3}c_{3}}\left(\frac{1+\gamma }{1-\gamma }\right),\;\mathrm{where}\;\gamma =\frac{{\left(\rho_{1}c_{1}+\alpha \rho_{2}c_{2}\right)}^{2}}{4\alpha \rho_{1}c_{1}\rho_{2}c_{2}}\frac{\sigma_{T3}}{\sigma_{I}}.$$

**Figure 19:**
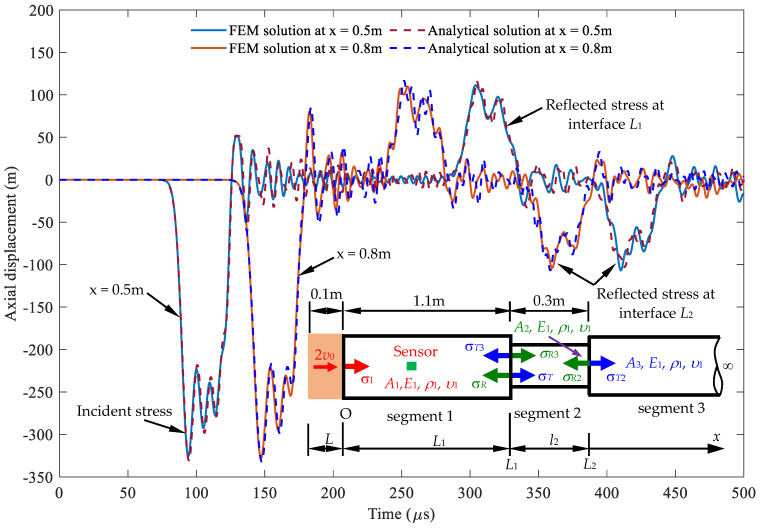
Comparison of stress wave propagation in segment 1 between the analytical solution and FEM results, observed at *x* = 0.5 m and *x* = 0.8 m.

To verify the signal received at the sensor location, determined by Equation (38), a comparison with the FEM is illustrated in Figure 19. The parameters of the material properties and geometries used are listed in Table 1. Additionally, there is a modification where *A*_1_ = *A*_3_ = 2*A*_2_ and the length *L* of the striker rod is 0.1 m. The results obtained indicate that there is good agreement between the analytical solution and the FEM results.

**Figure 20:**
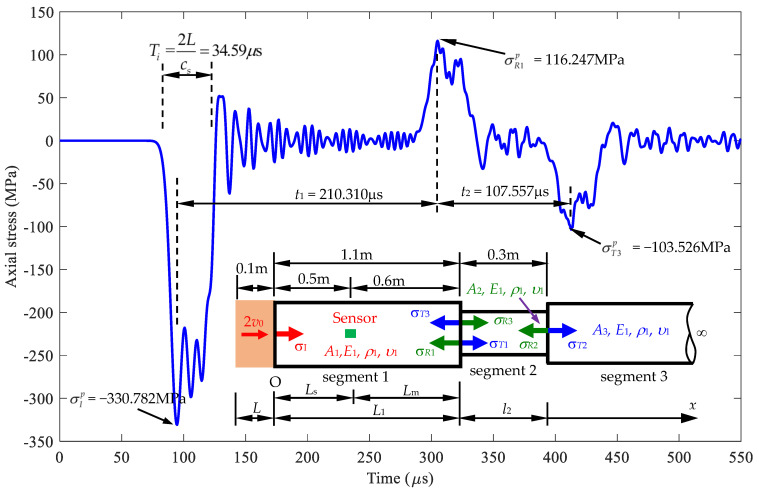
The cross-sectional area (*A*_2_, *A*_3_) and length (*L*_1_, *l*_2_) were determined based on the stress signal measured at *x* = *L*_s_.

**Figure 21:**
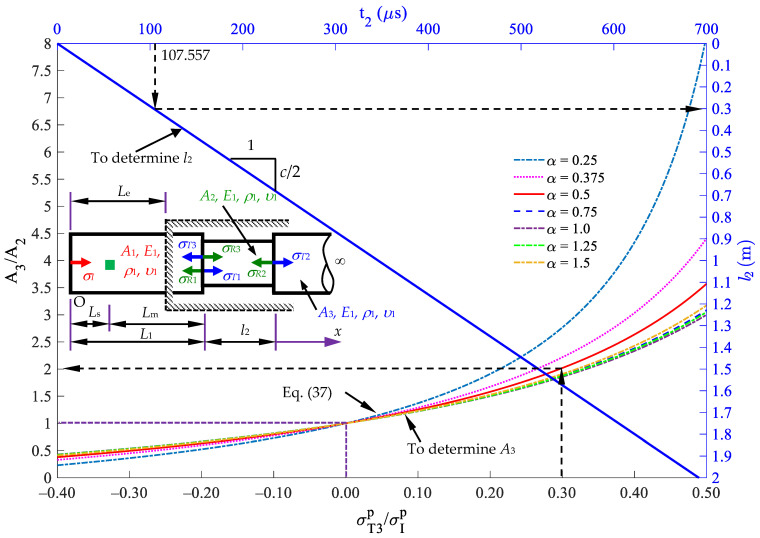
Diagram for determining cross-sectional area *A*_3_ and length *l*_2_ of $\alpha =A_{2}/A_{1}$.

The general steps of the processed NDT method to identify defect sizes in the rod (see Figure 20) are as follows:

1. First, use a striker rod with a length of *L* to impact the semi-infinite rod with an initial velocity of 2*v*_0_ to generate a stress wave propagating in the rod.
2. Place a strain sensor at position *x* = *L_s_* on the semi-infinite rod to receive the deformation signal generated by the striker rod.
3. The first part of the signal received at position *x* = *L_s_* is the incident stress, with a peak stress value of $\sigma_{I}^{p}$ and a duration of $T_{i}$. The wave then oscillates around zero.
4. The second part of the signal obtained is the reflected stress $\sigma_{R1}^{p}$ and a time interval from the first stress peak to the second stress peak of *t*_1_. From this signal, *A*_2_ and *L*_1_ can be determined as follows: the first peak incident stress is $\sigma_{I}^{p}$, and the first peak reflected stress is $\sigma_{R1}^{p}$, leading to a reflected ratio of $\sigma_{R1}^{p}/\sigma_{I}^{p}$. Figure 15 shows the corresponding cross-sectional area ratio $A_{2}/A_{1}=\alpha$ (or *A*_2_ = *αA*_1_). The received signal of the reflected stress at *t*_1_ corresponds to *L*_m_ in Figure 15. The location of the first cross-sectional area variation, *L*_1_, is calculated as *L*_m_ + *L*_s_.
5. The third part of the signal obtained is the reflected stress $\sigma_{T3}^{p}$ at a time interval *t*_2_ from the second stress peak to the third stress peak. From this signal, *A*_3_ and *L*_2_ can be determined as follows: the first peak reflected stress $\sigma_{T3}^{p}$ leads to a reflected ratio of $\sigma_{T3}^{p}/\sigma_{I}^{p}$. Based on Figure 21, the corresponding cross-sectional area ratio is $A_{3}/A_{2}=\beta$ with *A*_2_ = *αA*_1_ and $A_{3}=\alpha \beta A_{1}$. The received signal of the reflected stress at *t*_2_ corresponds to *l*_2_ in Figure 21.

**Figure 22:**
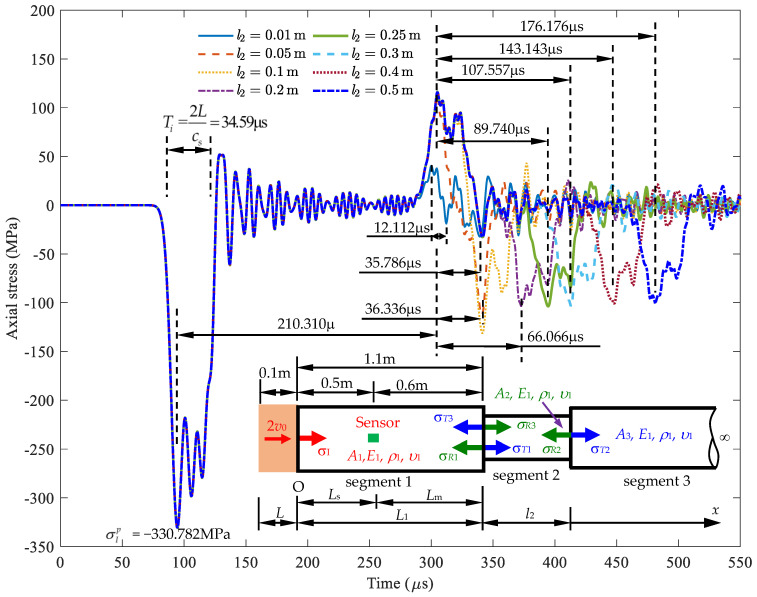
The signal received at the sensor location when the length *l*_2_ varies.

As shown in Figure 22 and Table 7, the signals are received at the sensor position with various length of *l*_2_ (area reduction zone). As the length of *l*_2_ increases, the time *t*_2_ to receive the reflected signal also increases. The main wavelength *λ* of the stress impulse of duration *T_i_* is equal to

(38) $$\lambda =cT_{i}=c\frac{2L}{c_{s}\;}=2L\left(\frac{c}{c_{s}\;}\right),$$

where *c* is the wave speed of the semi-infinite rod material and *c*_s_ is the wave speed of the striker rod material. 

From Equation (38), we know that the wavelength *λ* can be less than 2*L* when the striker rod material has a higher Young’s modulus than the semi-infinite rod material. When *c* = *c*_s_, the wavelength *λ* is equal to 2*L*. It is known from the NDT theory that the defect of size *l*_2_ can be identified only by wavelength *λ* if the impact wave is smaller than *l*_2_, i.e., *λ* = 2*L* < *l*_2_. The damaged length *l*_2_ is determined as

(39) $$l_{2}=\frac{1}{2}t_{2}c.$$

When *l*_2_ is less than or equal to 2*L* (2 × 0.1 m = 0.2 m), the received signal is noisy due to the effects of lateral inertia and Poisson’s ratio; this is indicated in Table 7 with a large error (>5%), making it difficult to accurately determine *l*_2_. This result highlights the significant influence of inertia and Poisson’s ratio on stress wave propagation in the rod, which causes noise in the signal when the damaged length *l*_2_ is small, thus complicating the determination of changes in length and cross-sectional area. From Equation (38), it is known that the wavelength *λ* of the detecting incident wave is affected by the striker rod length *L* and the wave speed ratio of the rod and striker materials. The minimum defect length *l*_2_ that can be identified is equal to the wavelength *λ* of the incident wave. The result is confirmed through the comparison in Table 7 of the calculated lengths (*L*_1_, *l*_2_) and cross-sectional areas (*A*_2_, *A*_3_) using the Rayleigh–Love theory with the real lengths and real cross-sectional areas.

The examples presented above aim to determine the change in cross-sectional area and its position in a rod based on the Rayleigh-Love rod theory. The model remains theoretical; it assumes the material to be linearly elastic, homogeneous, isotropic, and uniformly varying in cross-section, which may differ from real-world conditions. This theoretical work, however, establishes a foundation for understanding the behavior of stress wave propagation in Rayleigh–Love rods with abrupt variations in the cross-sectional area, considering lateral inertia and Poisson’s ratio. This understanding is crucial for developing accurate nondestructive testing methods applicable in real-world scenarios. Based on this research, further developments will be pursued with more complex material models and intricate geometric changes. This approach will enhance the applicability of our method for nondestructive testing (NDT) of rod-type structural elements under more realistic conditions.

## 5. Conclusions

This paper presents a detailed study of stress wave propagation in a Rayleigh–Love rod characterized by a sudden change in the cross-sectional area after a distance from the impacted end of the rod. Based on the analytical solution by Yang et al. [16], the analytical solutions of the transmitted and reflected stresses in a Rayleigh–Love rod with sudden cross-section variation were obtained. This study highlights the influence of a sudden cross-sectional variation on wave behavior, triggering reflections and transmissions at the interface of discontinuity. The role of Poisson’s effect is emphasized, demonstrating how it modulates wave behavior in the rod with discontinuities. Examples solved using the FEM verify the correctness of the modeling and numerical algorithm in terms of the analytical results of this study. 

In addition to the forward analysis of Rayleigh–Love wave propagation in rods impacted by a striker rod with the same impedance as the rod to be inspected, an impact-echo-type NDT method is proposed to assess the condition of rod-type structural components with sudden cross-sectional area changes within a cover medium based on the measured signal at the measurable zone of the rod to be inspected.

The investigation results show that when determining the cross-sectional area using the *σ_R_*/*σ_I_* and *σ*_*T*3_/*σ_I_* ratios, the decision to consider or not consider Poisson’s ratio has little influence on the final ratio (Figure 15 and Figure 21). Including Poisson’s ratio leads to a similar rate of increase in amplitudes in the incident and reflected stress waves. As a result, the reflected ratio $\sigma_{R}^{p}/\sigma_{I}^{p}$ remains nearly the same, regardless of whether Poisson’s ratio is accounted for or not. However, in practical applications, the first peak $\sigma_{I}^{p}$, $\sigma_{R}^{p}$, and $\sigma_{T3}^{p}$ values can be easily determined through measured signals. The technique proposed in this study can be easily applied in the field to conduct nondestructive evaluation of the location *L*_1_, extension *l*_2_, and ratios of cross-sectional area changes (*A*_2_/*A*_1_, *A*_3_/*A*_2_) of a defect in a rod within a cover medium.

## Figures and Tables

**Table 1:** The parameters of material properties and geometries of the striker rod and the segment rods used in example 1.

Parameters	Values
Diameter of the striker rod	30 mm
Diameter of segment 1	30 mm
Diameters of segment 2	30, 42, 52, 60, 67 mm
Young modulus, *E*	195 GPa
Poisson’s ratio, *υ*	0.3
Mass density, *ρ*	7850 kg/m^3^
Striker rod length, *L*	0.15 m
Length of segment 1, *L*_1_	1 m
Length of segment 2, *L*_2_	1.5 m
Impact velocity, 2*v*_0_	11.6 m/s
Wave speed of the semi-infinite rod material, *c*	5782.69 m/s
Wave speed of the striker rod material, *c*_s_	5782.69 m/s

**Table 2:** Reflected stresses in case of *A*_2_ ≥ *A*_1_.

*A*_2_/*A*_1_	$\sigma_{R}^{p}(\upsilon )$ (MPa)	$\sigma_{R}^{p}(\upsilon =0)$ (MPa)	Diff. (%)
1	0	0	-
2	−108.688	−87.762	23.84
3	−163.362	−131.643	24.09
4	−196.481	−157.972	24.38
5	−218.610	−175.524	24.55

**Table 3:** Transmitted stresses in case of *A*_2_ ≥ *A*_1_.

*A*_2_/*A*_1_	$\sigma_{T}^{p}(\upsilon )$ (MPa)	$\sigma_{T}^{p}(\upsilon =0)$ (MPa)	Diff. (%)
1	−333.24	−263.286	26.57
2	−222.16	−175.524	26.57
3	−166.62	−131.643	26.57
4	−133.296	−105.314	26.57
5	−111.08	−87.762	26.57

**Table 4:** Reflected stresses in case of *A*_2_ ≤ *A*_1_.

*A*_2_/*A*_1_	$\sigma_{R}^{p}(\upsilon )$ (MPa)	$\sigma_{R}^{p}(\upsilon =0)$ (MPa)	Diff. (%)
1	0	0	-
1/2	114.9129	87.762	30.94
1/3	170.8699	131.643	29.80
1/4	204.1672	157.9716	29.24
1/5	226.3654	175.524	28.97

**Table 5:** Transmitted stresses in case of *A*_2_ ≤ *A*_1_.

*A*_2_/*A*_1_	$\sigma_{T}^{p}(\upsilon )$ (MPa)	$\sigma_{T}^{p}(\upsilon =0)$ (MPa)	Diff. (%)
1	−333.240	−263.286	26.57
1/2	−444.320	−351.048	26.57
1/3	−499.860	−394.929	26.57
1/4	−533.184	−421.2576	26.57
1/5	−555.400	−438.81	26.57

**Table 6:** The initial impact kinetic energy $\left(1/2mv_{0}^{2}\right)$ of the striker in each case in Figure 17.

No.	Cross-Section (m^2^)	Density (kg/m^3^)	Impact Length (m)	Initial Velocity (m/s)	Kinetic Energy (J)
1	7.069 × 10^−4^	7850	0.15	2.9	3.500
2				5.8	14.000
3				11.6	55.999
4			0.075	2.9	1.750
5				5.8	7.000
6				11.6	27.999

**Table 7:** Comparison of calculated length (*L*_1_, *l*_2_) and cross-sectional areas (*A*_2_, *A*_3_) using Rayleigh–Love theory with real lengths and real cross-sectional areas.

No.	Length *L*_1_			Length *l*_2_			Cross-Section *A*_2_			Cross-Section *A*_3_		
	*L*_1calculated_ (m)	*L*_1real_ (m)	Error (%)	*l*_2calculated_ (m)	*l*_2real_ (m)	Error (%)	*A*_2calculated_ (cm^2^)	*A*_2real_ (cm^2^)	Error (%)	*A*_3calculated_ (cm^2^)	*A*_3real_ (cm^2^)	Error (%)
1	1.094	1.1	0.57	0.035	0.01	250.20	5.531	3.534	56.49	4.018	7.069	43.16
2	1.108	1.1	0.73	0.103	0.05	106.94	3.742	3.534	5.87	7.914	7.069	11.95
3	1.108	1.1	0.73	0.105	0.1	5.06	3.392	3.534	4.02	9.252	7.069	30.89
4	1.108	1.1	0.73	0.191	0.2	4.49	3.392	3.534	4.02	7.400	7.069	4.69
5	1.108	1.1	0.73	0.259	0.25	3.79	3.392	3.534	4.02	7.390	7.069	4.55
6	1.108	1.1	0.73	0.311	0.3	3.66	3.392	3.534	4.02	7.376	7.069	4.34
7	1.108	1.1	0.73	0.414	0.4	3.47	3.392	3.534	4.02	7.233	7.069	2.32
8	1.108	1.1	0.73	0.509	0.5	1.88	3.392	3.534	4.02	7.122	7.069	0.75

**Figure A1:**
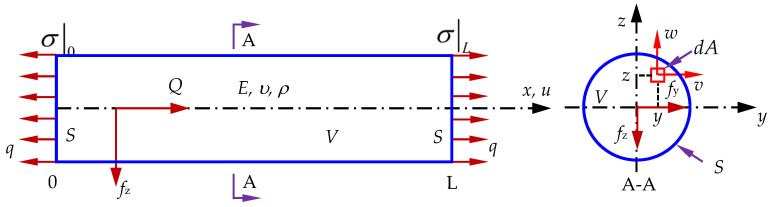
The rod and the cross-section illustrate the longitudinal and lateral displacement components due to Poisson’s effect.

## Data Availability

Data are contained within the article.

## Appendix A. Derivation of the Wave Velocity Taking into Account the Effect of Poisson’s Ratio

From the Navier–Cauchy equation of linear elasticity, we have

(A1) $$\rho \underset{˜}{\ddot{u}}=(\lambda +\mu )\underset{˜}{\nabla }(div\underset{˜}{u})+\mu \nabla^{2}\underset{˜}{u}.$$

Take the divergence of Equation (A1).

(A2) $$\underset{˜}{\nabla }\left(\rho \underset{˜}{\ddot{u}}\right)=(\lambda +\mu )\underset{˜}{\nabla }\left(\underset{˜}{\nabla }(div\underset{˜}{u})\right)+\underset{˜}{\nabla }\left(\mu \nabla^{2}\underset{˜}{u}\right),$$

(A3) $$\rho div\underset{˜}{\ddot{u}}=(\lambda +\mu )\nabla^{2}(div\underset{˜}{u})+\mu \nabla^{2}\left(div\underset{˜}{u}\right),\;\mathrm{where}\;\nabla^{2}=\underset{˜}{\nabla }\cdot \underset{˜}{\nabla },$$

(A4) $$\rho div\underset{˜}{\ddot{u}}=(\lambda +2\mu )\nabla^{2}(div\underset{˜}{u}),$$

(A5) $$∵div\underset{˜}{u}=\underset{˜}{\nabla }\cdot \underset{˜}{u}=\varepsilon_{kk}=\varepsilon_{11}+\varepsilon_{22}+\varepsilon_{33}.$$

Substituting Equation (A5) into Equation (A4), we obtain

(A6) $$(\lambda +2\mu )\nabla^{2}\varepsilon_{kk}=\rho_{0}{\ddot{\varepsilon }}_{kk},$$

And

(A7) $$\varepsilon_{kk}=\frac{\Delta V}{V},\;V_{1}=V+\Delta V=\left(1+\varepsilon_{kk}\right)V.$$

Based on the principle of mass conservation, we have

(A8) $$m=\rho_{1}V_{1}=\rho V.$$

Substituting Equation (A7) into Equation (A8), we obtain

(A9) $$\rho_{1}\left(1+\varepsilon_{kk}\right)V=\rho V.$$

(A10) $$\Rightarrow \rho_{1}=\frac{\rho }{\left(1+\varepsilon_{kk}\right)}=\rho \left(1-\varepsilon_{kk}\right),$$

where $\frac{1}{\left(1+\varepsilon_{kk}\right)}=1-\varepsilon_{kk}+\varepsilon_{kk}^{2}-\varepsilon_{kk}^{3}+\varepsilon_{kk}^{4}+\ldots \approx 1-\varepsilon_{kk}$.

From Equation (A10), we have

(A11) $$\rho_{1}-\rho =-\rho \varepsilon_{kk}.$$

Taking the derivative twice on both sides of Equation (A11), we obtain

(A12) $${\ddot{\rho }}_{1}=-\rho {\ddot{\varepsilon }}_{kk}.$$

In the same way, we obtain

(A13) $$\nabla^{2}\rho_{1}=-\rho \nabla^{2}\varepsilon_{kk}.$$

From Equations (A6), (A12) and (A13), we obtain

(A14) $$(\lambda +2\mu )\nabla^{2}\rho_{1}=\rho {\ddot{\rho }}_{1},$$

(A15) $$∴{\ddot{\rho }}_{1}=\frac{\left(\lambda +2\mu \right)}{\rho }\nabla^{2}\rho_{1}=c^{2}\nabla^{2}\rho_{1},$$

where

(A16) $$c=\sqrt{\frac{\lambda +2\mu }{\rho }}=\sqrt{\frac{(1-\upsilon )E}{(1+\upsilon )(1-2\upsilon )\rho }}$$

Equation (A15) describes motion in linear elasticity, where *c* is the wave velocity that takes into account the effect of Poisson’s ratio.

## Appendix B. Derivation of the Rayleigh–Love Equation of Stress Wave Propagation in the Rod

We consider a rod as shown in Figure A1, where *S* is the whole surface of the rod, *V* is the volume of the rod, and *Q*, *f*_y_, and *fz* are the components of the body force in the *x*, *y*, and *z* directions, respectively.

The strain–stress relationship from the general Hooke law is presented as

(A17) $$\begin{array}{l} \varepsilon_{ij}=\frac{1+\upsilon }{E}\sigma_{ij}-\frac{\upsilon }{E}\sigma_{kk}\delta_{ij}\\ \sigma_{ij}=\frac{E}{1+\upsilon }\varepsilon_{ij}+\frac{\upsilon E}{\left(1+\upsilon \right)\left(1-2\upsilon \right)}\varepsilon_{kk}\delta_{ij} \end{array},$$

where *E* is Young’s modulus, and *υ* is Poisson’s ratio of the rod material.

We assume that uniaxial stress still holds, so that $\sigma_{y}=\sigma_{z}=0$. Thus, Equation (A17) becomes

(A18) $$\varepsilon_{y}=\varepsilon_{z}=-\frac{\upsilon }{E}\sigma_{x}=-\upsilon \varepsilon_{x}=-\upsilon \frac{\partial u}{\partial x}.$$

The lateral displacements are determined as follows:

(A19) $$\begin{array}{cc} v=\varepsilon_{y}y, & w=\varepsilon_{z}z \end{array},$$

where *y* and *z* are the coordinates of a point in the cross-section. Substituting Equation (A18) into Equation (A19) enables the lateral displacements to be expressed in terms of the longitudinal motion as

(A20) $$\begin{array}{cc} v=-\upsilon y\frac{\partial u}{\partial x}, & w=-\upsilon z\frac{\partial u}{\partial x} \end{array}.$$

The expressions for the kinetic and potential energy are required in applying the energy method for the derivation of the equation of motion. The kinetic energy density is

(A21) $$T=\frac{\rho }{2}\left({\dot{u}}^{2}+{\dot{v}}^{2}+{\dot{w}}^{2}\right).$$

The strain energy density of this axially loaded rod is determined as follows:

(A22) $$V=\frac{1}{2}\sigma_{x}\varepsilon_{x},$$

Then, Hamilton’s equation becomes

(A23) $$\int_{t_{0}}^{t_{1}}\delta \tilde{W}dt+\delta \int_{t_{0}}^{t_{1}}\left(\tilde{T}-\tilde{V}\right)dt=0,$$

where

(A24) $$\begin{array}{l} \tilde{T}=\int_{V}\frac{\rho }{2}\left({\dot{u}}^{2}+{\dot{v}}^{2}+{\dot{w}}^{2}\right)dV\\ \tilde{V}=\int_{V}\frac{1}{2}\sigma_{x}\varepsilon_{x}dV\\ \tilde{W}={\tilde{W}}_{body}+{\tilde{W}}_{surface}=\int_{V}QudV+\int_{S}qudS \end{array},$$

where $\tilde{W}$ is the work carried out by the external forces, including *Q* as the body force and *q* as the surface force.

The preceding expressions can be integrated over the area of the section. From Equation (A20), we can obtain the following by considering the kinetic energy integral:

(A25) $$\begin{array}{cc} \dot{v}=-\upsilon y\frac{\partial^{2}u}{\partial x\partial t}, & \dot{w}=-\upsilon z\frac{\partial^{2}u}{\partial x\partial t} \end{array}.$$

Then, the following is obtained by substituting Equation (A25) into Equation (A24):

(A26) $$\tilde{T}=\int_{0}^{L}dx\int_{A}\frac{\rho }{2}\left({\dot{u}}^{2}+\upsilon^{2}\left(y^{2}+z^{2}\right){\left(\frac{\partial^{2}u}{\partial x\partial t}\right)}^{2}\right)dA.$$

The longitudinal displacement *u*(*x*,*t*) does not depend on the coordinates *y* and *z*. The second moment of area of a rod is determined as

(A27) $$I=\int_{A}\left(y^{2}+z^{2}\right)dA=\left(y^{2}+z^{2}\right)A.$$

Thus, the area integral may be evaluated exactly. The result is

(A28) $$\tilde{T}=\int_{0}^{L}\frac{\rho A}{2}\left\{{\dot{u}}^{2}+\upsilon^{2}k^{2}{\left(\frac{\partial^{2}u}{\partial x\partial t}\right)}^{2}\right\}dx=\int_{0}^{L}\frac{\rho A}{2}\left\{{\left(\frac{\partial u}{\partial t}\right)}^{2}+\upsilon^{2}k^{2}{\left(\frac{\partial^{2}u}{\partial x\partial t}\right)}^{2}\right\}dx.$$

where *k* is the radius of gyration of the cross-section; *k* is determined as

(A29) $$k^{2}=y^{2}+z^{2}=I/A.$$

where *I* is the second moment of area, and *A* is the cross-section area of the rod.

The potential energy density is determined as follows:

(A30) $$\tilde{V}=\int_{0}^{L}dx\int_{A}\frac{1}{2}\left(E\varepsilon_{x}\right)\varepsilon_{x}dA=\int_{0}^{L}dx\int_{A}\frac{1}{2}E\varepsilon_{x}^{2}dA=\int_{0}^{L}\frac{EA}{2}\varepsilon_{x}^{2}dx=\int_{0}^{L}\frac{EA}{2}{\left(\frac{\partial u}{\partial x}\right)}^{2}dx.$$

The only stress that exists here is *σ*_x_. Thus, only the body and surface forces compatible with *σ* exist. Moreover, only the axial body force *Q* and the end applied tractions *σ*(0,*t*) and *σ*(*L*,*t*) exist. Thus, we obtain

(A31) $$\delta \tilde{W}=\delta \left(\int_{V}QudV+\int_{A}\sigma udA\right)=\int_{V}Q\delta udV+\int_{A}\sigma \delta udA=A\left(\int_{0}^{L}Q\delta udx+\sigma \delta u{|}_{0}^{L}\right).$$

The variation in the kinetic and potential energy is

(A32) $$\delta I=\delta \int_{t_{0}}^{t_{1}}\left(\tilde{T}-\tilde{V}\right)dt=\delta \left(\int_{t_{0}}^{t_{1}}dt\int_{0}^{L}A\left[\frac{\rho }{2}\left\{{\left(\frac{\partial u}{\partial t}\right)}^{2}+\upsilon^{2}k^{2}{\left(\frac{\partial^{2}u}{\partial x\partial t}\right)}^{2}\right\}-\frac{EA}{2}{\left(\frac{\partial u}{\partial x}\right)}^{2}\right]dx\right).$$

We obtain the following through the procedure of the calculus of variation method:

(A33) $$\begin{array}{l} \int_{t_{0}}^{t_{1}}\int_{0}^{L}\rho \left[c_{0}^{2}\frac{\partial^{2}u}{\partial x^{2}}-\frac{\partial^{2}u}{\partial t^{2}}+\upsilon^{2}k^{2}\frac{\partial^{4}u}{\partial x^{2}\partial t^{2}}+\frac{Q}{\rho }\right]\delta udxdt-\int_{t_{0}}^{t_{1}}\left[\left(E\frac{\partial u}{\partial x}-\sigma +\rho \upsilon^{2}k^{2}\frac{\partial^{3}u}{\partial x\partial t^{2}}\right)\delta u\right]{|}_{0}^{L}dt\\ +\int_{0}^{L}\rho \left[\frac{\partial u}{\partial t}+\rho \upsilon^{2}k^{2}\frac{\partial^{3}u}{\partial x^{2}\partial t}\right]\delta u{|}_{t_{0}}^{t_{1}}dx=0 \end{array},$$

where $c_{0}=\sqrt{E/\rho }$.

We can obtain the following by applying the calculus of variation analysis:

(A34) $$\int_{t_{0}}^{t_{1}}\int_{0}^{L}\rho \left[c_{0}^{2}\frac{\partial^{2}u}{\partial x^{2}}-\frac{\partial^{2}u}{\partial t^{2}}+\upsilon^{2}k^{2}\frac{\partial^{4}u}{\partial x^{2}\partial t^{2}}+\frac{Q}{\rho }\right]\delta udxdt-\int_{t_{0}}^{t_{1}}\left[\left(E\frac{\partial u}{\partial x}-\sigma +\rho \upsilon^{2}k^{2}\frac{\partial^{3}u}{\partial x\partial t^{2}}\right)\delta u\right]{|}_{0}^{L}dt=0$$

According to variation theory, each expression within the square brackets of the first and second terms in Equation (A34) must be zero, and body force *Q* must be neglected. These latter conditions lead to Rayleigh–Love’s equation of motion.

(A35) $$\frac{\partial^{2}u}{\partial t^{2}}=c_{0}^{2}\frac{\partial^{2}u}{\partial x^{2}}+\upsilon^{2}k^{2}\frac{\partial^{4}u}{\partial x^{2}\partial t^{2}}$$
